# Synthesis and In Vitro Assessment of pH-Sensitive Human Serum Albumin Conjugates of Pirarubicin

**DOI:** 10.3390/ph14010022

**Published:** 2020-12-30

**Authors:** Kenji Tsukigawa, Shuhei Imoto, Keishi Yamasaki, Koji Nishi, Toshihiko Tsutsumi, Shoko Yokoyama, Yu Ishima, Masaki Otagiri

**Affiliations:** 1 Faculty of Pharmaceutical Sciences, Sojo University, 4-22-1 Ikeda, Nishi-ku, Kumamoto 860-0082, Japan; tsukigawa@ph.sojo-u.ac.jp (K.T.); simoto@ph.sojo-u.ac.jp (S.I.); kcyama@ph.sojo-u.ac.jp (K.Y.); knishi@ph.sojo-u.ac.jp (K.N.); 2 DDS Research Institute, Sojo University, 4-22-1 Ikeda, Nishi-ku, Kumamoto 860-0082, Japan; 3 School of Pharmaceutical Sciences, Kyushu University of Health and Welfare, 1714-1 Yoshino-machi, Nobeoka, Miyazaki 882-8508, Japan; t-tsutsumi@phoenix.ac.jp (T.T.); s.yokoyama@phoenix.ac.jp (S.Y.); 4 Department of Pharmacokinetics and Biopharmaceutics, Institute of Biomedical Sciences, Tokushima University, 1-78-1 Sho-machi, Tokushima 770-8505, Japan; ishima.yuu@tokushima-u.ac.jp

**Keywords:** pirarubicin (THP), human serum albumin (HSA), HSA-drug conjugates, pH-sensitive, drug release, cytotoxicity

## Abstract

In a previous study, we reported on the development of a synthetic polymer conjugate of pirarubicin (THP) that was formed via an acid-labile hydrazone bond between the polymer and the THP. However, the synthetic polymer itself was non-biodegradable, which could lead to unexpected adverse effects. Human serum albumin (HSA), which has a high biocompatibility and good biodegradability, is also a potent carrier for delivering antitumor drugs. The objective of this study was to develop pH-sensitive HSA conjugates of THP (HSA-THP), and investigate the release of THP and the cytotoxicity under acidic conditions in vitro for further clinical development. HSA-THP was synthesized by conjugating maleimide hydrazone derivatives of THP with poly-thiolated HSA using 2-iminothiolane, via a thiol-maleimide coupling reaction. We synthesized two types of HSA-THP that contained different amounts of THP (HSA-THP2 and HSA-THP4). Free THP was released from both of the HSA conjugates more rapidly at an acidic pH, and the rates of release for HSA-THP2 and HSA-THP4 were similar. Moreover, both HSA-THPs exhibited a higher cytotoxicity at acidic pH than at neutral pH, which is consistent with the effective liberation of free THP under acidic conditions. These findings suggest that these types of HSA-THPs are promising candidates for further development.

## 1. Introduction

Pirarubicin (4′-*O*-tetrahydropyranyldoxorubicin, THP), a semi-synthetic derivative of doxorubicin [1], shows much more rapid intracellular uptake, more effective antitumor activity, and lesser cardiac toxicity, compared to doxorubicin [2,3,4,5]. However, free THP, as well as free doxorubicin, is indiscriminately distributed to both tumor and normal healthy tissues.

Biocompatible macromolecular drugs (>40 kDa) show prolonged blood circulation and accumulate in tumor tissues preferentially [6,7,8].Thus, liposomes, polyethylene glycol (PEG) conjugates, and poly(*N*-(2-hydroxypropyl) methacrylamide) (PHPMA) conjugates, have been widely used as antitumor drug carriers.

It is also known that macromolecular drugs exhibit insufficient therapeutic effects due to the low release of the active form of the free drug from the macromolecular drug in the tumor tissues [9,10,11,12]. To address this limitation, we recently developed PHPMA conjugates of THP via the formation of a hydrazone bond resulting in a more effective release of free THP in the acidic milieu of tumor tissues [13,14,15,16].

However, PHPMA itself is a non-biodegradable synthetic polymer, and its use could lead to unexpected adverse effects, if the polymers were to be retained in the body for long periods. Lack of biodegradability would likely limit the use as drug carrier for cancer treatment. Human serum albumin (HSA), the most abundant protein in blood plasma, which has emerged as a potent carrier for improving the accumulation of drugs in tumors, has a high biocompatibility and good biodegradability [17,18,19,20,21,22,23,24,25].

We therefore developed HSA conjugates of THP (HSA-THP) with an acid cleavable hydrazone bond between HSA and THP for further development. We synthesized poly-thiolated HSA (HSA-thiol or HSA-SH) using 2-iminothiolane spacer for conjugation with THP. The conjugation of THP to HSA-SH was carried out using maleimide hydrazone derivatives of THP (THP-EMCH) via thiol-maleimide coupling reaction. In this study, we prepared two types of HSA-THP samples with different amounts of THP, and investigated the release of THP from the HSA-THPs and the cytotoxicity of the HSA-THPs under normal and at acidic pH conditions in vitro.

## 2. Results

### 2.1. Synthesis of THP-EMCH

**Figure 1:**
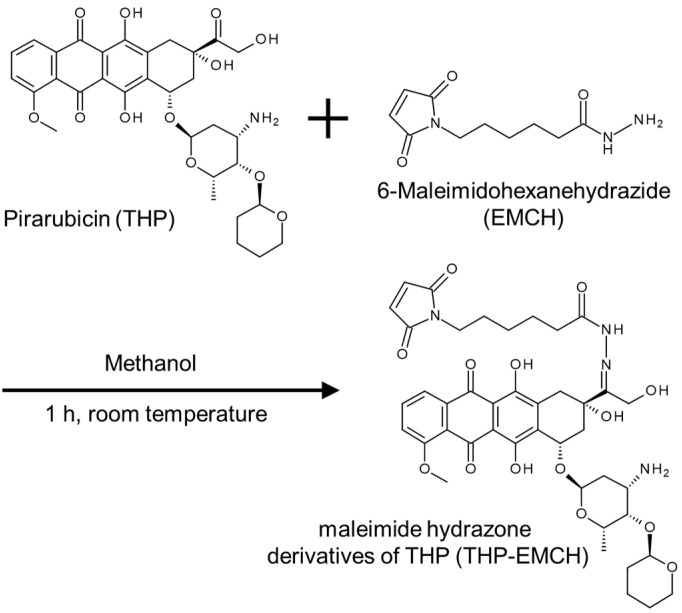
Synthesis of maleimide hydrazone derivatives of pirarubicin (THP) (THP-EMCH) using 6-maleimidohexanehydrazide (EMCH). Chemical structures and conjugation pathway.

**Figure 2:**
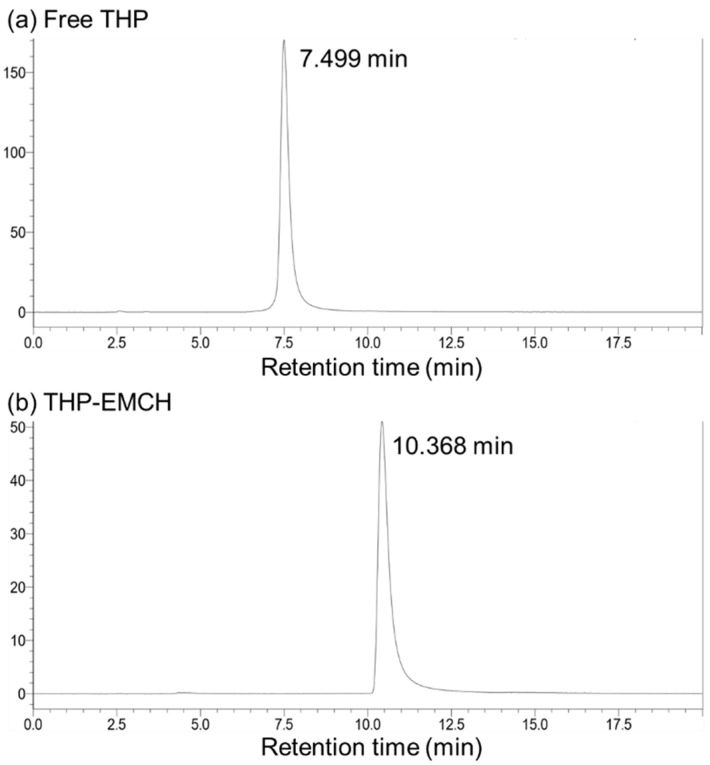
HPLC analyses of (**a**) free THP and (**b**) THP-EMCH. HPLC was performed on a Shimadzu HPLC system equipped with an RF-10AXL fluorescence detector (excitation at 488 nm, emission at 590 nm). See text for detail.

From 100 mg of THP and 108 mg of EMCH, 110 mg of THP-EMCH was obtained (Figure 1). HPLC analysis showed that the THP-EMCH preparation contained no free unconjugated THP or decomposition product (Figure 2a,b). The structure of THP-EMCH was confirmed by ^1^H-NMR, ^13^C-NMR, and MS (ESI). ^1^H-NMR (500 MHz, DMSO-D6) δ 10.29 (s, 1H), 7.91–7.93 (m, 2H), 7.65–7.67 (m, 1H), 6.99–7.00 (m, 2H), 5.73 (t, *J* = 4.6 Hz, 1H), 5.54 (s, 1H), 5.32 (d, *J* = 2.9 Hz, 1H), 4.95 (t, *J* = 6.9 Hz, 1H), 4.50–4.51 (m, 1H), 4.39 (ddd, *J* = 23.2, 14.3, 4.9 Hz, 2H), 4.07–4.09 (m, 1H), 3.99 (s, 3H), 3.91–3.94 (m, 1H), 3.78 (s, 1H), 3.38–3.46 (m, 1H), 3.20–3.24 (m, 2H), 2.55–2.50 (m, 1H), 2.04–2.22 (m, 3H), 1.74–1.91 (m, 4H), 1.44 (m, 6H), 1.26–1.31 (m, 3H), 1.18–1.19 (m, 3H), 0.99–1.02 (m, 2H). ^13^C-NMR (126 MHz, DMSO-D6) δ 186.79, 186.71, 174.03, 171.15, 161.04, 156.69, 154.36, 152.46, 136.84, 136.52, 135.92, 134.96, 134.59, 120.17, 120.02, 119.22, 110.85, 110.73, 101.14, 99.06, 73.21, 72.76, 72.47, 66.29, 65.13, 64.48, 56.81, 56.14, 47.01, 36.89, 33.96, 31.43, 30.73, 29.06, 27.95, 26.02, 24.98, 23.58, 21.00, 17.70, 15.39. MS (ESI): *m*/*z* calcd for C_42_H_51_N_4_O_14_ [M+H]^+^ 835.3402, found 835.3373.

### 2.2. Synthesis of HSA-THP2 and HSA-THP4

**Figure 3:**
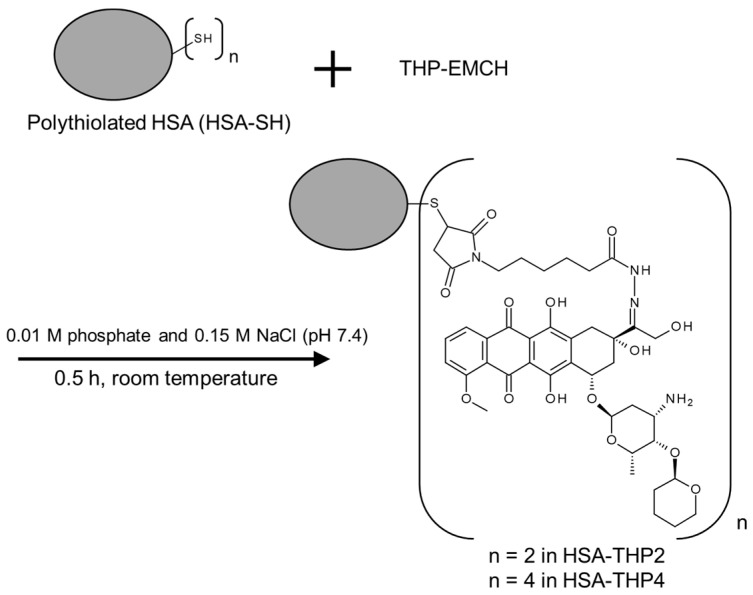
Synthesis of HSA-THP2 and HSA-THP4.

**Figure 4:**
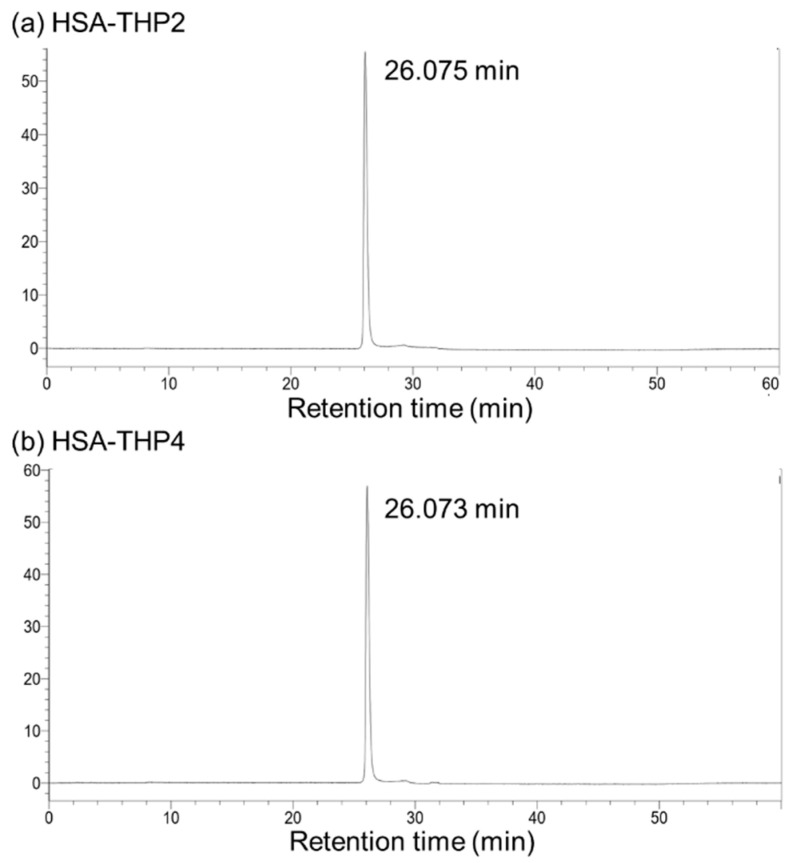
HPLC analyses of (**a**) HSA-THP2 and (**b**) HSA-THP4. HPLC was performed on a Shimadzu HPLC system equipped with an RF-10AXL fluorescence detector (excitation at 488 nm, emission at 590 nm). See text for detail.

As shown in Figure 3, we synthesized two types of HSA-THP that contained different amounts of conjugated THP molecules by using different concentrations of THP-EMCH in the reactions. The HSA-THP2 sample contained approximately two THP molecules (2.03 ± 0.12 mol THP/mol of HSA). Moreover, the HSA-THP4 contained approximately four THP molecules (4.12 ± 0.14 mol THP/mol HSA). HPLC analyses of HSA-THP2 and HSA-THP4 showed that neither free THP nor decomposition products were detected in the product (Figure 4a,b).

The water solubility of both of the HSA-THP samples was higher than that for free THP. The average sizes of the HSA-THP2 and HSA-THP4 in aqueous solution were determined to be 7.4 nm with a polydispersity index (PDI) of 0.016 and 7.7 nm with a PDI of 0.030, respectively. There was no significant difference in size between the normal HSA (7.7 nm with a PDI of 0.006) and the HSA-THPs. The zeta potential of the HSA-THP2 and HSA-THP4 samples were determined to be −22.82 mV and −20.15 mV, respectively.

**Figure 5:**
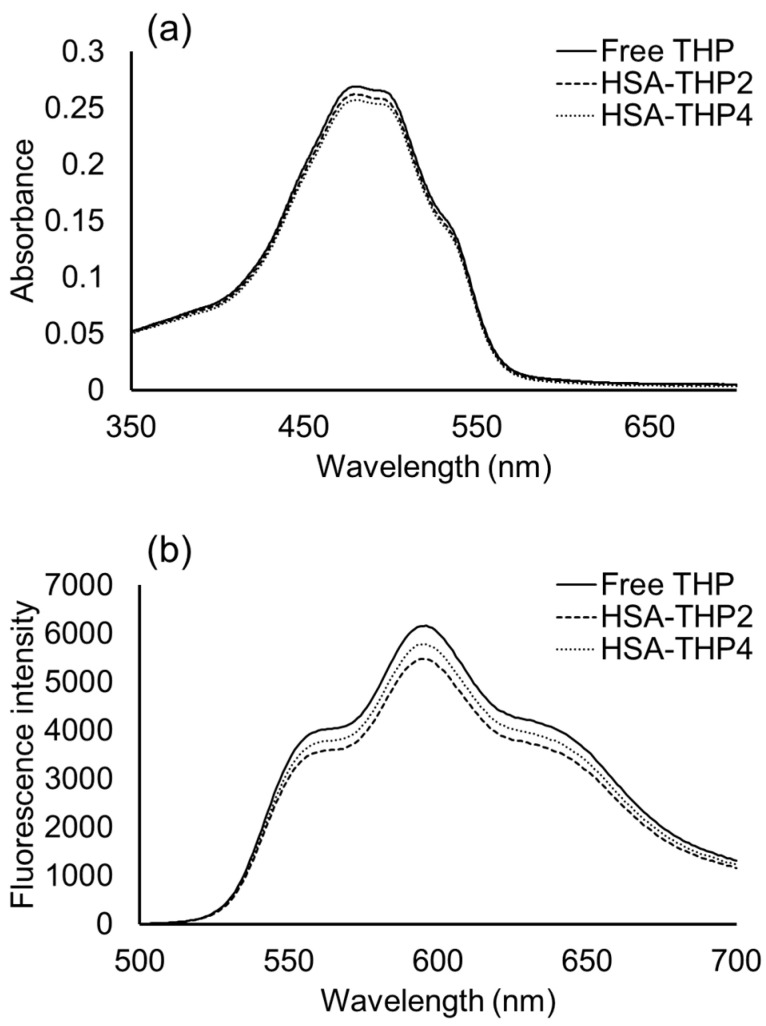
(**a**) UV/VIS spectra and (**b**) fluorescence spectra of HSA-THP2 and HSA-THP4 in 0.01 M phosphate and 0.15 M NaCl (pH 7.4).

Figure 5 shows UV/VIS spectra and fluorescence spectra of HSA-THP2 and HSA-THP4 in 0.01 M phosphate and 0.15 M NaCl (pH 7.4). No significant change was observed between the free THP and HSA-THPs, indicating the absence of any π–π stacking interactions between THP molecules that were bound to HSA.

### 2.3. Release of Free THP from HSA Conjugates under Acidic pH Conditions

**Figure 6:**
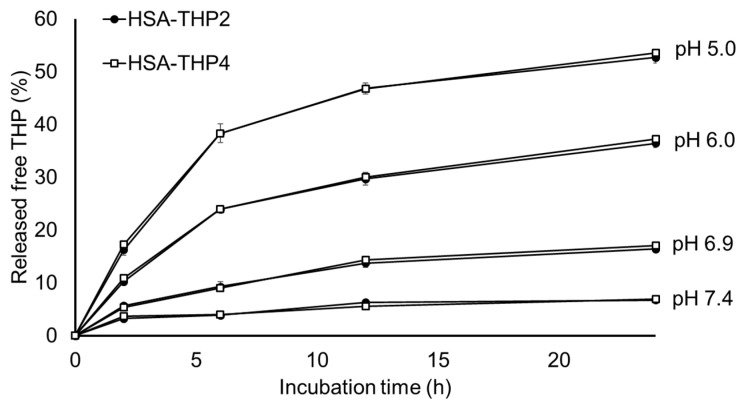
Release of free THP from HSA-THP at different pH values. Incubation was at 37 °C for the indicated time periods. The amount of released free THP was measured by means of HPLC. Values are the means ± S.E. (*n* = 5).

THP was conjugated to HSA via an acid-labile hydrazone linkage in both HSA-THPs. We examined the behavior of the HSA-THPs at different pH values. As expected, free THP was released more efficiently at an acidic pH (Figure 6). The percent release of THP from HSA-THP2 was 6.8% at pH 7.4, 16.5% at pH 6.9, 36.4% at pH 6.0, and 52.7% at pH 5.0 in 24 h. The percent release of THP from HSA-THP4 was 7.0% at pH 7.4, 17.1% at pH 6.9, 37.3% at pH 6.0, and 53.5% at pH 5.0 in 24 h. The rate of release of free THP from either of the HSA conjugates was essentially the same.

### 2.4. In Vitro Cytotoxicity of HSA-THP2 and HSA-THP4

**Figure 7:**
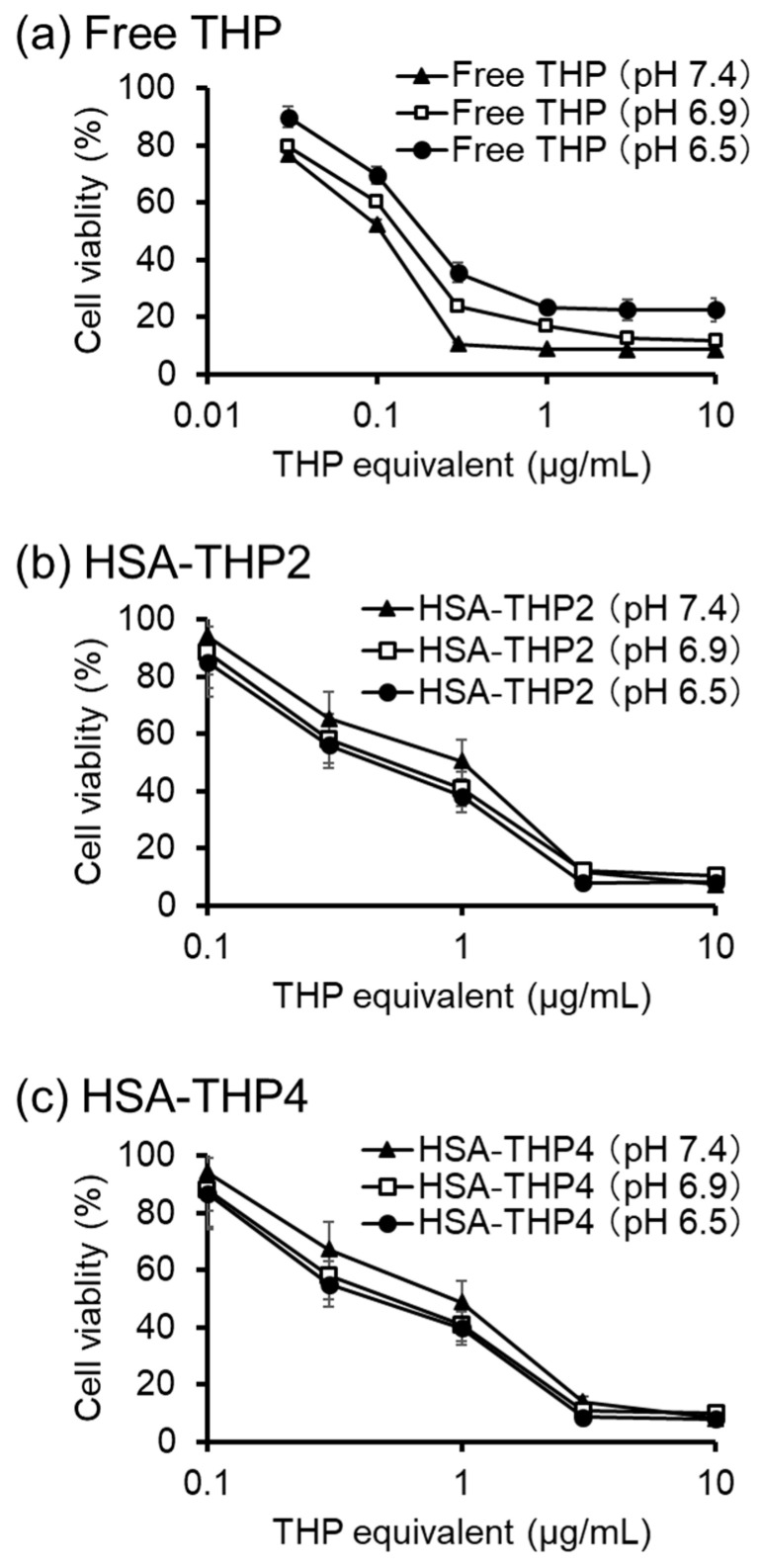
In vitro cytotoxicity of (**a**) free THP, (**b**) HSA-THP2, and (**c**) HSA-THP4 against HeLa cells at different pH values (pH 7.4, pH 6.9, pH 6.5). Cells were treated with THP derivatives for 48 h, followed by an MTS Assay to quantify the numbers of viable cells. Values are the means ± S.E. (*n* = 6).

The cytotoxicity of the HSA-THP2 and HSA-THP4 samples was investigated using HeLa cells at physiological pH 7.4, and at pH 6.9 and pH 6.5 approximate pH values for tumor tissue (Figure 7, Table 1). After 48 h incubation, the half-maximal inhibitory concentration (IC_50_) values of free THP at pH 7.4, 6.9, and 6.5 were 0.11 ± 0.01, 0.15 ± 0.01, and 0.22 ± 0.02 μg/mL respectively (Figure 7a). IC_50_ values of HSA-THP2 at pH 7.4, 6.9, and 6.5 were 1.01 ± 0.09, 0.65 ± 0.06, and 0.55 ± 0.04 μg/mL respectively (Figure 7b). IC_50_ values of the HSA-THP4 sample at pH 7.4, 6.9, and 6.5 were 1.01 ± 0.12, 0.63 ± 0.06, and 0.54 ± 0.04 μg/mL respectively (Figure 7c). Both HSA conjugates showed a more potent cytotoxicity at pH 6.9 and pH 6.5 than at pH 7.4. We also found no significant difference in cytotoxicity between the HSA-THP2 and HSA-THP4 samples.

**Figure 8:**
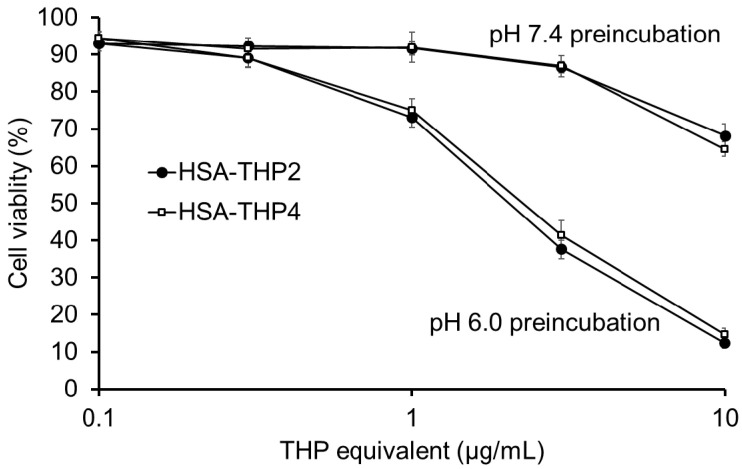
In vitro cytotoxicity of HSA-THP2 or HSA-THP4 against HeLa cells after preincubation in solutions at different pH values. HSA-THP2 or HSA-THP4 was incubated in different buffers pH 7.4 or pH 6.0 for 18 h before being exposed to HeLa cells for 3 h, followed by 48 h of culture. After treatment, MTS Assay was performed to quantify viable cells. Values are the means ± S.E. (*n* = 6).

We then investigated the relationship between the release of free THP from the HSA conjugates and in vitro cytotoxicity. HSA-THP2 and HSA-THP4 samples were preincubated in different buffers at pH 7.4 or pH 6.0 in the absence of HeLa cells for 18 h and were then applied to HeLa cells for 3 h, followed by a 48 h culture period (Figure 8). The results showed that the preincubation of HSA-THP2 and HSA-THP4 at pH 6.0 resulted in a dramatic enhancement in their cytotoxicity, and no significant difference in cytotoxicity was found between HSA-THP2 and HSA-THP4.

## 3. Discussion

HSA is an excellent carrier for drug delivery because it is biodegradable and lacks toxicity and immunogenicity. Moreover, our previous studies indicated that the synthesis of polymers, such as PHPMA, is a complicated, time-consuming and expensive process [13]. These circumstances prompted us to develop the HSA based antitumor drug THP delivery system in this study for further clinical development.

We previously developed an HSA based delivery system that contained numerous conjugated nitric oxide units by chemical modification [23]. In the present work, we prepared HSA-THP with two or more conjugated THP units by using a procedure similar to that for poly-*S*-nitrosated HSA above (Figure 1 and Figure 3). In these HSA based delivery carriers, 2-iminothiolane, which reacts with primary amines to introduce SH groups, was used as the thiolation reagent of HSA. Other studies have shown that poly-thiolated albumin prepared by other methods resulted in the formation of aggregates as a result of intermolecular disulfide formation [26,27].

Acidic pH sensitive linkages such as hydrazone bonds and acetal bonds between a carrier and an antitumor drug are widely utilized for the effective liberation of a free drug in the acidic environment of tumor tissues or lysosomes [13,14,28,29,30,31,32,33]. The findings concerning the release of a drug from HSA-THPs reported herein also showed that free THP is released more rapidly at acidic pH than at neutral pH (Figure 6). Therefore, although we performed the experiment for only 24 h, we considered that the purpose of the pH-sensitive assay for the THP releasability of HSA-THP was achieved. The drug release studies for a longer period will be needed for further discussion of drug release and cytotoxicity in future. We also found no significant difference in the percent release of THP between HSA-THP2 and HSA-THP4. These results suggest that the amount of THP attached to HSA may not affect the sensitivity to hydrolysis of hydrazone bond.

The UV/VIS spectra and fluorescence spectra of HSA-THP2 and HSA-THP4 indicated that there was no π–π stacking interactions between THP molecules that were bound to HSA (Figure 5). In addition, there was no significant difference in size between normal HSA and HSA-THPs, as evidenced by dynamic light scattering (DLS) analyses. These results suggest the absence of an association of HSA-THP.

We also investigated the cytotoxicity of free THP and the HSA-THPs at different pH values (pH 7.4, 6.9, and 6.5), with pH 7.4 representing normal tissue and pH 6.5 and pH 6.9 representing tumor tissue. The HSA-THP samples exhibited a higher cytotoxicity against HeLa cells at an acidic pH (pH 6.9 and pH 6.5) than at a neutral pH. Moreover, preincubation of HSA-THPs at pH 6.0, which is one of the pH representing tumor tissue, enhanced its cytotoxicity. The higher cytotoxicity at an acidic pH may be due to the release of THP from the HSA conjugate and the subsequent cellular uptake of the released THP. Further studies of cytotoxicity with preincubation at various pHs (e.g., pH 6.9, pH 6.5 and pH 5.0) and of the intracellular uptake of HSA-THP are warranted.

In our previous study, the cytotoxicity of a polymer-THP conjugate which has non-cleavable linkage between the polymer and THP was less than 1/100 compared with that of free THP at pH 7.4 [34]. THP exerts cytotoxic activity mainly by inhibiting DNA synthesis via the intercalation into nucleotide bases. These findings strongly support the importance of drug release from macromolecular drugs for achieving efficient antitumor activity. Meanwhile, the slow release of free THP at neutral pH would reduce the adverse effect of THP. Further investigations including animal experiments are clearly warranted.

This is the first report on the preparation and characterization of poly THP conjugated HSA via an acid-labile hydrazone linkage as an anticancer nanomedicine. We conclude that HSA-THPs appear to be promising candidates for serving as biocompatible macromolecular antitumor drugs for further development.

## 4. Materials and Methods

### 4.1. Materials

Pirarubicin (4′-*O*-tetrahydropyranyldoxorubicin, THP) was purchased from Selleck Biotech (Tokyo, Japan). 6-Maleimidohexanehydrazide trifluoroacetate (EMCH) was purchased from Tokyo Chemical Industry Co., Ltd. (Tokyo, Japan). 2-iminothiolane was from Thermo Fisher Scientific (Tokyo, Japan). Human serum albumin (HSA) was purchased from the Japan Blood Products Organization (Tokyo, Japan) and defatted by means of a charcoal treatment [35]. Methanol (Super Dehydrated), diethyl ether, dimethyl sulfoxide (DMSO), acetonitrile, penicillin G, streptomycin, were purchased from Wako Pure Chemical (Osaka, Japan). Dulbecco’s Modified Eagle Medium (DMEM) was purchased from Nissui Seiyaku (Tokyo, Japan). Fetal calf serum was obtained from GIBCO (Grand Island, NY, USA). Diethylenetriaminepentaacetic acid (DTPA) and 5,5′-dithiobis(2-nitrobenzoic acid) (DTNB) were purchased from Dojindo Chemical Laboratories (Kumamoto, Japan). A CellTiter 96^®^ Aqueous One solution containing 3-(4,5-dimethylthiazol-2-yl)-5-(3-carboxy-methoxyphenyl)-2-(4-sulfophenyl)-2*H*-tetrazolium (MTS) was from Promega (Madison, WI, USA). All chemicals were used without further purification.

### 4.2. Synthesis of Maleimide Hydrazone Derivatives of THP (THP-EMCH)

THP-EMCH was synthesized by reacting THP and EMCH (Figure 1). THP (100 mg, 159.3 µmol) and EMCH (108 mg, 318.7 µmol) were dissolved in 5 mL of methanol and the resulting mixture was stirred 1 h at room temperature in the dark. The reaction was monitored by HPLC as described below. After the reaction, products were precipitated with diethyl ether, and washed with methanol and diethyl ether and then dried in vacuum. The THP-EMCH was obtained as a red solid (108 mg, 129.3 µmol, 81% yield).

### 4.3. Synthesis of Poly-Thiolated HSA (HSA-SH)

Terminal SH groups were added to the HSA molecule by incubating 0.15 mM HSA in a 100 mM potassium phosphate buffer containing 0.5 mM DTPA, pH 7.8, at room temperature with 6 mM 2-iminothiolane for 0.5 h, as described previously [23]. Unreacted 2-iminothiolane was removed by ultrafiltration with a molecular weight cut-off of 30,000 Da. HSA-SH was obtained as a powder by lyophilization. The amount of SH groups in the HSA-SH was quantified using the DTNB method. A standard curve of reduced glutathione was used for the determination of the amount of SH groups. The number of SH groups attached to HSA-SH was estimated to be 4.62 ± 0.39 mol (*n* = 5).

### 4.4. Synthesis of HSA Conjugates of THP (HSA-THP)

In this study, two types of HSA-THP conjugates with different amounts of THP were synthesized. For the synthesis of HSA-THP with a low THP load in the conjugate, 0.4 mM THP-EMCH and 0.15 mM HSA-SH in 0.01 M phosphate and 0.15 M NaCl (pH 7.4) was reacted for 0.5 h at room temperature (Figure 3). After the reaction, unreacted THP-EMCH was removed by ultrafiltration with a molecular weight cut-off of 30,000 Da, and the resulting solution was lyophilized. The THP content in HSA-THPs was quantified spectrophotometrically (V-530; JASCO, Tokyo, Japan) by the absorbance of THP at 480 nm. A standard curve for free THP in DMSO was used as the reference. The number of THP units in the HSA-THP was estimated to be 2.03 ± 0.12 mol (*n* = 5). We denote the HSA-THP with the lower drug load as HSA-THP2.

HSA-THP with a higher THP load in the conjugate was synthesized by a procedure similar to that for HSA-THP2 except that 0.8 mM THP-EMCH was used. The number of THP units in the HSA-THP was estimated to be 4.12 ± 0.14 mol (*n* = 5). We denote this HSA-THP with a higher THP load as HSA-THP4.

### 4.5. In Vitro Drug Release from HSA-THPs

The objective of this drug release analysis was to confirm THP release in response to acidic environment. In this study, the free THP that was released from the HSA-THPs was extracted from the incubation solution by chloroform, and the released free THP was then detected and quantified by HPLC. Each of the HSA-THP samples were dissolved at a concentration of 5.0 mg/mL in 0.1 M sodium acetate buffer (pH 5.0) or 0.1 M phosphate buffer (pH 6.0, pH 6.9, pH 7.4) and incubated at 37 °C. After the indicated times, an aliquot of the solution was mixed with an equal volume of 0.2 M sodium bicarbonate buffer (pH 9.8) and three times the volume of chloroform to extract the released free THP from HSA-THPs into the chloroform. The chloroform phase was evaporated to dryness, and the pellet was dissolved in the HPLC mobile phase and analyzed by a Shimadzu HPLC system. A standard curve of free THP was used for determination of the amount of released free THP. The percent release of free THP relative to the total amount of THP bound to HSA in HSA-THP conjugate was calculated.

### 4.6. ^1^H-NMR, ^13^C-NMR, ESI-Mass, Dynamic Light Scattering (DLS), Zeta Potential, Fluorescence Spectroscopy, and HPLC

^1^H-NMR and ^13^C-NMR spectra were obtained in DMSO-D6 with a JEOL ECA 500 NMR (JEOL Ltd., Tokyo, Japan) spectrometer at 500 and 126 MHz, respectively.

ESI-mass spectral analyses were performed on a JEOL JMS-T100LP system (JEOL Ltd., Tokyo, Japan).

Each HSA-THP sample was dissolved in 0.01 M phosphate and 0.15 M NaCl (pH 7.4) at a concentration of 2.0 mg/mL and filtered through a 0.2 µm filter. Hydrodynamic size and surface charge (zeta potential) were measured by light scattering (ELS-Z2; Photal Otsuka Electronics, Osaka, Japan).

Each HSA-THP sample was dissolved in 0.01 M phosphate and 0.15 M NaCl (pH 7.4), and fluorescence spectra were recorded with a Shimadzu RF-6000 fluorescence spectrophotometer with excitation at 488 nm and emissions between 500 and 700 nm.

HPLC was performed on a Shimadzu HPLC system equipped with an RF-10AXL fluorescence detector (excitation at 488 nm, emission at 590 nm).

For the analysis of THP-EMCH by HPLC, an Inertsil WP300 C18 (4.6 mm × 150 mm, GL Sciences, Tokyo, Japan) column was used, and the column temperature was maintained at 40 °C. The mobile phase consisted of 35% acetonitrile and 65% 20 mM sodium phosphate buffer (pH 7.0) at flow rate of 1.0 mL/min.

For the analysis of HSA-THP by HPLC, an Inertsil WP300 C18 column (4.6 mm × 150 mm, GL Sciences, Tokyo, Japan) was used, and the column temperature was maintained at 40 °C. The gradient mobile phase A consisted of 35% acetonitrile and 65% 20 mM sodium phosphate buffer (pH 7.0), and mobile phase B consisted of 70% acetonitrile and 30% 20 mM sodium phosphate buffer (pH 7.0): 0–20 min 100% mobile phase A; 20–25 min increase to mobile phase B; 25–45 min 100% mobile phase B; 45–50 min decrease to mobile phase A; 50–60 min 100% mobile phase A. Flow rate was 1.0 mL/min.

For measuring the release of THP from HSA-THP by HPLC, the column was a COSMOSIL 5C_8_-MS (4.6 mm × 150 mm) (Nacalai Tesque, Kyoto, Japan), and the column temperature was maintained at 40 °C. The mobile phase consisted of 33% acetonitrile and 67% 0.1 M sodium acetate buffer (pH 5.0) at flow rate of 1.2 mL/min. A standard curve for free THP was used for the determination of the amount of free THP that was released.

### 4.7. In Vitro Cytotoxicity Assay

In this study, to compare cytotoxicity at a normal pH and an acidic pH, normal HeLa cells were cultured in medium adjusted to pH 7.4, and the acid-adapted HeLa cells continuously cultured (>1 month) in medium adjusted to pH 6.9 or pH 6.5. It was difficult to adapt tumor cells to below pH 6.5 in vitro. Therefore, in vitro cytotoxicity assays were performed at pH 7.4, pH 6.9, and pH 6.5. Thus, all experiments were performed on growing cells at each pH. HeLa cells were maintained in DMEM supplemented with 10% fetal calf serum under 5% CO_2_/95% air at 37 °C. Three types of DMEM with different pH values (pH 6.5, pH 6.9, and pH 7.4), prepared by adding different amounts of NaHCO_3_ (0.5 g/L, 1.0 g/L and 3.7 g/L, respectively), were used in this study. Cells (3000 cells/well) were plated in 96-well plates (TrueLine, La Crosse, WI, USA). After an overnight incubation, free THP, HSA-THP2, or HSA-THP4 was added, followed by changing to fresh medium after a 48 h culture to remove the added samples in culture medium, and then an MTS Assay to quantify viable cells with an absorbance at 490 nm by using Varioskan LUX (Thermo Fisher Scientific, Waltham, MA, USA).

To investigate the relationship between the drug release from the HSA conjugates and cytotoxicity, HSA-THP2 and HSA-THP4 were preincubated in different buffers at pH 7.4 or pH 6.0 in the absence of HeLa cells for 18 h and were then applied to HeLa cells for 3 h, followed by a 48 h culture period. Cell viability was determined by MTS assay, as described above.

HSA is considered to show no effect on assays in this study, since there have been no reports on the interaction between HSA and MTS regents and cytotoxicity of HSA.

### 4.8. Statistical Analysis

Data are presented as the mean ± S.E. To determine the significance of the results obtained, the two-tailed unpaired Student’s *t*-test was applied. Results were considered statistically significant when *p* was <0.05.

## Figures and Tables

**Table 1:** Half-maximal inhibitory concentration (IC_50_) values of free THP, HSA-THP2, and HSA-THP4 against HeLa cells after a 48 h drug exposure.

IC_50_ (µg/mL THP Equivalent)	Free THP	HSA-THP2	HSA-THP4
pH 7.4	0.11 ± 0.01	1.01 ± 0.09	1.01 ± 0.12
pH 6.9	0.15 ± 0.01	0.65 ± 0.06 *	0.63 ± 0.06 *
pH 6.5	0.22 ± 0.02	0.55 ± 0.04 *	0.54 ± 0.07 *

Values are the means ± S.E. (*n* = 6). * *p* < 0.05, significant differences from IC_50_ value at pH 7.4 for each drug.

## Data Availability

The data presented in this study are available in this article.
